# The Tubulin Inhibitor VERU-111 in Combination With Vemurafenib Provides an Effective Treatment of Vemurafenib-Resistant A375 Melanoma

**DOI:** 10.3389/fphar.2021.637098

**Published:** 2021-03-25

**Authors:** Hongmei Cui, Qinghui Wang, Duane D. Miller, Wei Li

**Affiliations:** ^1^ Department of Pharmaceutical Sciences, University of Tennessee Health Science Center, Memphis, TN, United States; ^2^ Institute of Toxicology, School of Public Health, Lanzhou University, Lanzhou, China

**Keywords:** VERU-111, vemurafenib-resistance, melanoma, ERK, akt, skp2

## Abstract

Melanoma is one of the deadliest skin cancers having a five-year survival rate around 15–20%. An overactivated MAPK/AKT pathway is well-established in BRAF mutant melanoma. Vemurafenib (Vem) was the first FDA-approved BRAF inhibitor and gained great clinical success in treating late-stage melanoma. However, most patients develop acquired resistance to Vem within 6–9 months. Therefore, developing a new treatment strategy to overcome Vem-resistance is highly significant. Our previous study reported that the combination of a tubulin inhibitor ABI-274 with Vem showed a significant synergistic effect to sensitize Vem-resistant melanoma both *in vitro* and *in vivo*. In the present study, we unveiled that VERU-111, an orally bioavailable inhibitor of α and β tubulin that is under clinical development, is highly potent against Vem-resistant melanoma cells. The combination of Vem and VERU-111 resulted in a dramatically enhanced inhibitory effect on cancer cells *in vitro* and Vem-resistant melanoma tumor growth *in vivo* compared with single-agent treatment. Further molecular signaling analyses demonstrated that in addition to ERK/AKT pathway, Skp2 E3 ligase also plays a critical role in Vem-resistant mechanisms. Knockout of Skp2 diminished oncogene AKT expression and contributed to the synergistic inhibitory effect of Vem and VERU-111. Our results indicate a treatment combination of VERU-111 and Vem holds a great promise to overcome Vem-resistance for melanoma patients harboring BRAF (V600E) mutation.

## Introduction

Melanoma is one of the most common skin cancers, and the five-year survival rate for metastatic melanoma is 15–20% (Patel et al., 2020). Exposure to UV radiation increases the risk of DNA damage and genetic changes, thus confers susceptibility to melanoma.

It is well-established that the mitogen-activated protein kinase (MAPK) and phosphatidylinositol 3-kinase (PI3K)/protein kinase B (AKT) signaling pathways are overactivated in melanoma since BRAF mutation leads to uncontrollable cell growth and ultimately develops into cancer (Lim et al., 2017; Faghfuri et al., 2018). BRAF mutant melanoma accounts for nearly 50% of metastatic melanoma cases, among which V600E mutant represents 84.6% of the BRAF mutations (Patel et al., 2020). Currently, targeted therapies for metastatic melanoma mainly include BRAF and MEK inhibitors, such as Vemurafenib (the first FDA-approved BRAF inhibitor), dabrafenib, encorafenib, trametinib (the first FDA-approved MEK inhibitor), cobimetinib, and binimetinib (Shirley 2018). However, although ATP-competitive BRAF (V600E) kinase inhibitor such as Vem or its combination with a MEK inhibitor has dramatically improved the treatment outcome for patients with metastatic melanoma (Spain et al., 2016; Simeone et al., 2017; Trojaniello et al., 2019), over 50% of patients develop acquired drug resistance and began to show signs of tumor recurrence within 6–9 months of treatment (Torres-Collado et al., 2018).

Several mechanisms have been documented to mediate Vem-resistance, for example, overexpression of P-glycoprotein (P-gp), BRAF mutation, aberrant expression of miRNA, translocation of E3 ligase, or PI3K/AKT pathway (Johnson et al., 2014; Duggan et al., 2017; Lim et al., 2017; Thang et al., 2017; Diaz-Martinez et al., 2018). Such mechanistic understandings have led to a number of exciting synergistic combinations to re-sensitize Vem against metastatic melanoma. For example, JQ1, a bromodomain inhibitor, was found to re-sensitize the Vem-resistant melanoma cells to undergo apoptosis *in vitro* by decreasing the expressions of P-gp and acetylated histone H3 (Zhao et al., 2018). Since checkpoint kinase 1 (Chk1) plays a pivotal role in controlling cell cycle progression, Hwang et al. reported that PF477736 (a potent and specific inhibitor of Chk1) effectively promotes Vem-resistant melanoma cells to regain sensitivity to Vem by lowering the total level of Chk1 and modifying its phosphorylation (Hwang et al., 2018). Recently, PRIMA-1^Met^, also known as APR-246, propels both Vem-sensitive and Vem-resistant melanoma cells to apoptotic cell death via directly activating p53 and indirectly inhibiting PI3K/AKT pathway (Krayem et al., 2016).

**FIGURE 1:**
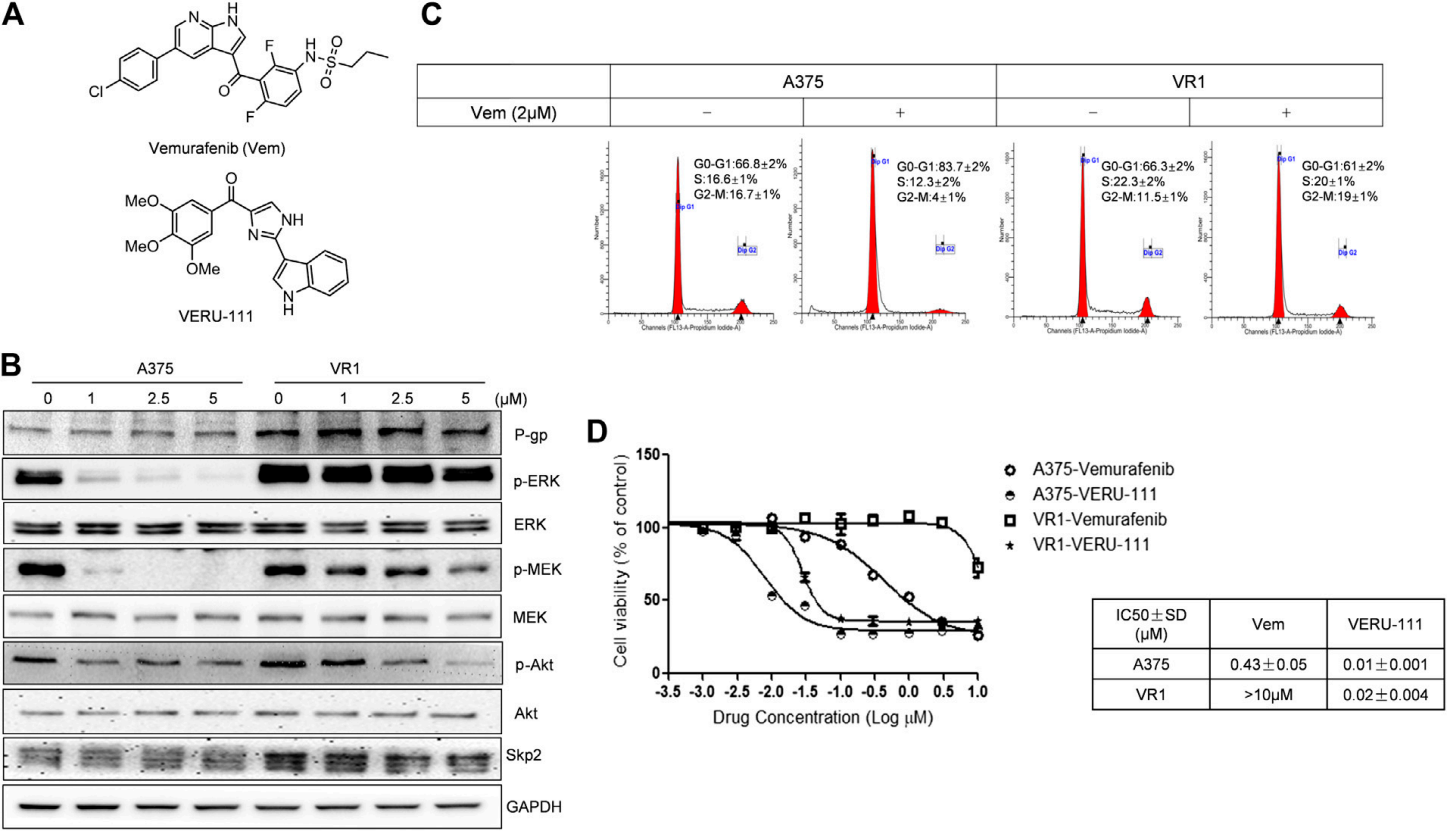
VERU-111 is efficient in both A375 and A375-Vem resistant (VR1) cells **(A)** Structure of compound Vemurafenib (Vem) and VERU-111 **(B)** p-ERK/p-MEK is persistantly expressed in VR1 cells **(C)** Vem treatment leads to G0-G1 arrest in both parental A375 and Vem-resistant VR1 cell **(D)** MTS assay to test IC_50_ in parental A375 melanoma and VR1 cells.

Although combinations of BRAF inhibitor with MEK or ERK inhibitors benefit Vem-resistant patients (Atefi et al., 2011; Gadiot et al., 2013; Pulkkinen et al., 2020), half of the patients still gain resistance after 6–8 months. Intriguingly, some tubulin destabilizing agents reported previously by us targeting the colchicine-binding site showed promise to overcome Vem-resistance, paclitaxel-resistance in melanoma, breast cancer, lung cancer, prostate cancer, cervical cancer et al. (Wang et al., 2014; Guan et al., 2017; Arnst et al., 2018; Deng et al., 2020; Kashyap et al., 2020; Mahmud et al., 2020). In our previous studies, we have demonstrated that a tool tubulin inhibitor, ABI-274, showed strong synergistic efficacy in a Vem-resistant xenograft mouse model (Wang et al., 2014). Besides, the combination of Vem with ABI-274 arrested both A375 and A375 Vem-resistant cells at both G0-G1 and G2-M phase, which arrested the tumor cells at G2-M phase and captured resistant cells escaping from G0-G1 Phase. ABI-274 is a tool compound developed in our lab as a potent tubulin inhibitor that binds to the colchicine binding site (Chen et al., 2010; Wang et al., 2018). Further structural optimization from ABI-274 led to VERU-111 (Figure 1A), which is orally available and much more potent and less toxic in several types of tumor models, including prostate cancer, melanoma, breast cancer, lung cancer, and pancreatic cancer (Chen et al., 2012; Deng et al., 2020; Kashyap et al., 2020; Mahmud et al., 2020). As literature reported, the IC_50_ of ABI-274 was 25.3 nM while IC_50_ of VERU-111 was 8.2 nM in MDA-MB-231 breast cancer cell, and the IC_50_ of ABI-274 was 18.7 nM while IC_50_ of VERU-111 was 10.4 nM in A375 melanoma cell (Chen et al., 2020). VERU-111 has now been under Phase 1b/2 clinical trials for men with metastatic castration and androgen-blocking agent resistant prostate cancer (ClinicalTrials.gov Identifier: NCT03752099) and holds great promise to become an oral tubulin inhibitor targeting the colchicine binding site. In the present study, we investigated the ability of VERU-111 to re-sensitize Vem and thus effectively overcome Vem-resistance in BRAF^V600E^ melanoma tumor models.

## Materials and Methods

### Reagents and Cell Lines

Vemurafenib was purchased from LC Laboratories (Woburn, MA), and VERU-111 was synthesized as described previously (Figure 1A). The human melanoma A375 cell line was acquired from ATCC (ATCC^®^ CRL-1619) and maintained in DMEM with 10% FBS. Vemurafenib-resistant melanoma cells were built according to literature (Su et al., 2012). Briefly, cells were chronically selected by culturing A375 cells in increasing concentrations of Vem for at least 3 months and named VR1 cells. The isolated resistant VR1 cell line steadily increased IC_50_ values for Vem above 10 μm and maintained in full growth medium containing 5 μm Vem. VR1-SgSkp2 cells generated from VR1 cells and Skp2 was knocked out with guide RNA sequence: 5′-atg​cac​agg​aag​cac​ctc​c-3′, screened with puromycin and maintained in full growth medium containing 5 μm Vem.

### Cell Proliferation and IC_50_ Measurement

Cell proliferation was determined using the MTS [3-(4,5-dimethylthiazol-2-yl)-5(3-carboxymethoxyphenyl)-2-(4-sulfopheny)-2H-tetrazolium, inner salt] reagents (Promega, Madison, WI) following manual instruction. Briefly, cells were seeded at a concentration of 5,000 cells/well in 96-well plate, on next day, the cell culture medium contains the vemurafenib or VERU-111 at different concentrations was added into the well with four duplications. After 72 h later, 20 ul MTS solution was added and measured at 490 nm absorbance. IC_50_ was calculated using Graphpad Prism software with transformed drug concentration in Log10. Compound concentrations used *in vivo* animal study was based on previous publication (Wang et al., 2014).

### Cell-Cycle and Apoptosis Analysis

To determine apoptosis and cell-cycle distributions, treated cells (24 h) were harvested with trypsin and fixed in 70% cold ethanol for overnight, then stained with PI (50 μg/ml)/RNase (100 μg/ml) solution for 60 min at room temperature in the dark according to the manufacturer’s instructions (Sigma Aldrich, St. Louis, MO). Cell apoptosis was monitored by using the Annexin V-FITC Apoptosis Detection Kit (Abcam) following manufacturer’s instructions, and the data was processed using the Modfit 2.0 software and analyzed by a BD LSR-II cytometer (BD Biosciences).

### Colony Formation Assays

For colony formation assays, 1,000 cells were plated in 6-well plates with triplicates, compound with indicated concentration was added in the next day, and surviving colonies were stained with crystal violet 10 days later and counted.

### Western Blot Analysis

At the indicated time (24 h), treated A375, VR1, VR1-SgSkp2 cells were collected to investigate levels of relevant cascade protein or apoptotic markers by Western blot analysis. The following antibodies from Cell Signaling were used: p-ERK1/2 (#9101), p44/42 MAPK (ERK1/2; #9102), p- AKT (Ser473; #9271), AKT (#9272), cleaved PARP (#9185), or GAPDH (#3683). Skp2 antibody was purchased from Santa Cruz (sc-74477).

### Endogenous Co-Immunoprecipitation Assay

For the co-immunoprecipitation assay, A375 cells and VR1 cells were treated with 5 μm Vem for 24 h, the cell lysates were incubated with A/G beads (Millipore) with corresponding equal amount of antibody in RIPA buffer (50 mM Tris-HCl, pH 7.4, 100 mM NaCl, 1% NP-40, 0.1% SDS, 0.5% sodium deoxycholate and 1 mM EDTA) at 4°C overnight. After extensive washes, precipitated proteins on beads were boiled and loaded onto SDS-PAGE gel and further performed Western blotting.

### Vemurafenib-Resistant Tumor Xenograft and Treatment

6–8-weeks NSG male mice were provided by Dr Seagrous lab. VR1 cells were suspended in PBS and mixed with high concentration Matrigel (BD Biosciences) at a ratio of 2:1 right before use. 100 μl of this mixture containing 2 × 10^6^ cells were injected subcutaneously to the right-side dorsal flank of each mouse. The regimen formulation and treatment refer to (Wang et al., 2014). Briefly, VERU-111 or Vem was diluted in PEG300 (Sigma Aldrich) and administered through intraperitoneal injection once per day, 5 days per week for three continuous weeks. Tumor volume and body weight of each mouse were measured three times per week. At the end of the experiments, mice were euthanized and tumor tissues were isolated and prepared for pathogen analysis. One-way ANOVA was used to compare tumor size and body weight for *in vivo* xenograft study. Tumor growth inhibition (TGI) was calculated as 100 − 100 × [(T − T0)/(C − C0)], and tumor regression was calculated as (T − T0)/T0 × 100, where T, T0, C, and C0 are the mean tumor volume for the specific group on the last day of treatment, mean tumor volume of the same group on the first day of treatment, mean tumor volume for the vehicle control group on the last day of treatment, and mean tumor volume for the vehicle control group on the first day of treatment, respectively (Wang et al., 2014).

### Pathology and Immunohistochemistry Analysis

Tumor tissues fixed in formalin buffer for more than 1 week were stained with hematoxylin and eosin (H&E). For immunohistochemistry (IHC) analysis, the excised tumor tissues were collected in 10% formalin and embedded in paraffin. The following primary antibodies were used with rabbit anti-Ki67 (#9027, Cell Signaling Technology), rabbit anti-cleaved-caspase 3 (#9664, Cell Signaling Technology), rabbit anti-phospho-ERK1/2 (#4376, Cell Signaling Technology), rabbit anti-AKT (#4691, Cell Signaling Technology), p-AKT (#4060) following HRP-DAB-methods with signal boost reagents (#8114, Cell Signaling Technology). Slides were imaged with BZ-X700 microscope and analyzed by image J.

### Statistical Analysis

Data were analyzed using Prism Software 5.0 (GraphPad Software, Inc.). The statistical significance (*p* < 0.05) was evaluated by student t test, and one-way ANOVA.

## Results

### Development of Vem-Resistant VR1 Cells From Vem-Sensitive A375 Cells

A375 cells are one of the most widely used and representative V600E mutant melanoma cells, and we have previously reported the anti-tumor efficiency of VERU-111 in many cancer types (Kashyap et al., 2019; Deng et al., 2020; Kashyap et al., 2020; Mahmud et al., 2020) as well as the synergy of Vem in combination with ABI-274 in A375 Vem-resistant melanoma cells (Wang et al., 2014). Herein, we investigate whether the combination of VERU-111 (ABI-274 derivative) with Vem can also overcome Vem-resistance in A375. A375 cells are one of the most widely used and representative V600E mutant melanoma cells. We first developed Vem-resistant cells (VR1) from the BRAF^V600E^ mutant A375 melanoma cells by increasing the concentration of Vem as reported previously (Su et al., 2012). As expected, the persistent expressions of p-ERK, p-MEK and overexpression of P-gp were detected in VR1-Vem treatment cells (Figure 1B), the hallmarks of acquired Vem-resistance (Boussemart et al., 2014). AKT has no significant change in VR1 cells, accompanied with decreased AKT activation (p-AKT) in a Vem-dependent manner. Notably, when treated with Vem at 2 μm, A375 cells but not resistant VR1 cells were effectively arrested at G0-G1 phase (Figure 1C). Furthermore, the IC_50_ value of Vem in VR1 cells (>10 μm) increased more than 25-fold compared with that in the parental A375 cells (0.43 μm, Figure 1D). All these together strongly support the Vem-resistant property of VR1 cells. In contrast, the IC_50_ value of VERU-111 in VR1 cells only marginally increased from 0.01 to 0.02 μm, which is in line with our previous result, indicating the ability of VERU-111 to overcome Vem-resistance as a single agent (Wang et al., 2014).

### Combination of VEM with VERU-111 Inhibits Cell Proliferation and Increases Apoptosis in Both A375 and VR1 cells by Inhibiting AKT Expression

**FIGURE 2:**
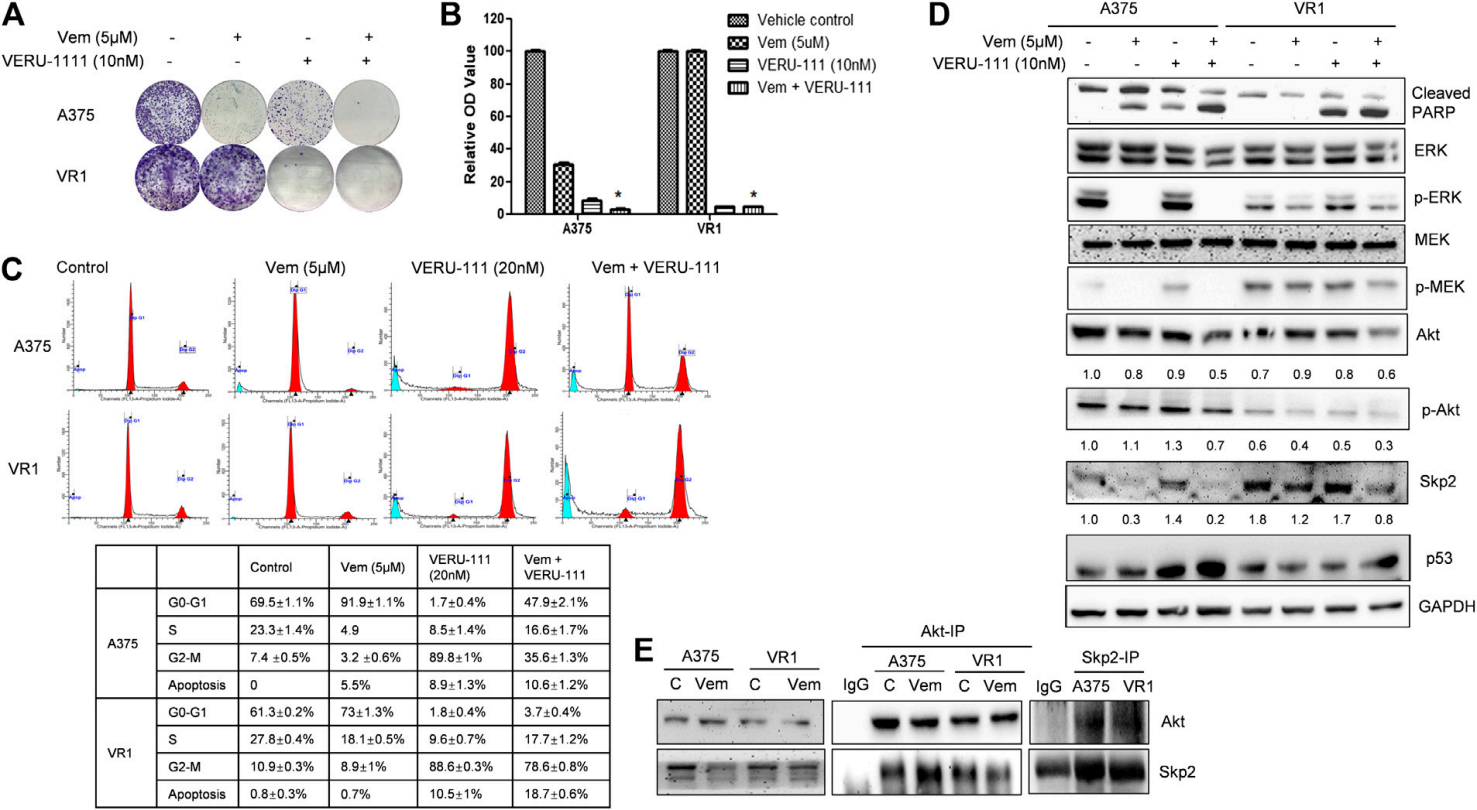
Combination treatment of Vem and VERU-111 sensitizes both A375 and VR1 cells **(A)** Colony formation assay. 1,000 Cells were seeded in the 12-well plates and treated with indicated concentration of drugs, after 8 days, stained with crystal violet **(B)** Quantification of clone formation assay. Colonies were lyzed in buffer with SDS, then read the absorbance at 490 nm *, *p* < 0.05, compared with single-agent treatment groups **(C)** Cell cycle distribution and apoptosis of combination of vemurafenib and VERU-111 in A375 and VR1 cells. Data was analyzed with Modfit 2.0 software, and apoptosis cells were counted automatically **(D)** Signaling pathway in both A375 and VR1 cells after corresponding treatment **(E)** Both A375 and VR1 cell were treated with DMSO and 5uM Vem, and then performed endogenous Co-IP between AKT and Skp2.

Next, we investigated whether VERU-111 has any synergistic interaction with Vem on melanoma cell lines, by comparing the single-agent treatment efficacy with their combination in both A375 and VR1 cells. Colony formation assays unveiled that proliferation of both parental A375 cells and Vem-resistant VR1 cells were inhibited following the entire regimen (Figures 2A,B). Moreover, Vem did not change cell cycle distribution of both cell lines, while addition of tubulin inhibitor bypassed G0-G1 cell cycle phase and arrested cell cycle at G2-M phase in both A375 and VR1 cells (Figure 2C). As Figure 2C showed that in VR1 cells, there is 61.3, 27.8, 10.9% of cells distributing in the G0-G1, S or G2-M phase, respectively. Vem single treatment produced similar cell - cycle phase distribution. In VERU-111 single treatment group, the percentage of cells distributed in the G2-M phase had accumulated up to 88.6%. The combination of Vem and VERU-111 strongly arrested VR1 cells in both G0-G1 (3.7%) and G2-M (78.6%) phases while the combination regimen arrested parental A375 cells in both G0-G1 (47.9%) and G2-M (35.6%) phases, which indicated VERU-111 could capture Vem-resistant cells leaking from G0-G1 arrest, and thus produce a strong synergistic effect with Vem. Correspondingly, there are 0.8, 0.7, 10.5, 18.5% of apoptotic cells were detected in DMSO, Vem, VERU-111, combination treatment groups in VR1 cells respectively, and 0, 5.5, 8.9, 10.6% of apoptotic cells were observed in the indicated treatment groups in A375 cells. All these data suggested combination regimen has stronger efficiency in arresting the cell cycle and inducing apoptosis than a single treatment.

VR1 has sustained expressions of p-ERK upon single-agent Vem treatment (Figure 2D), similarly, sustained p-MEK expression was noted in VR1 cells after Vem treatment, consistent with the cross-resistance to MEK inhibitors in these Vem-resistant VR1 cells compared with the parental A375 cells (Figure 2D). In contrast, when treated with the combination of Vem and VERU-111, both A375 and VR1 cells had significantly more apoptosis (cleaved-PARP, Figure 2D), together with additional decreased expression of AKT expression and p-AKT activation (Figure 2D). In VR1 cells, the combination of VERU-111 and Vem reduced the level of AKT to 67 and 75% (0.6/0.9 × 100%, 0.6/0.8 × 100%) compared with single treatment, whereas the p-AKT expression level inhibited to 75 and 60% (0.3/0.4 × 100%, 0.3/0.5 × 100%) compared with a single treatment (Figure 2D). AKT is a serine/threonine kinase activated downstream of PI3K, which is a receptor for various pro-proliferation and bioactive substances. To our knowledge, the activation of AKT often contributes to tumorigenesis and plays a role in regulating cell motility, local invasion, and metastasis. Furthermore, our previously published outcomes proved that the synergistic anti-proliferation might be mediated by simultaneously targeting both ERK and AKT pathways (Wang et al., 2014).

Recently, F-box protein S-phase kinase-associated protein 2 (Skp2) was reported to be involved in drug resistance, including paclitaxel resistance(Kajiyama et al., 2007; Yang et al., 2014; Yang et al., 2016; Huang et al., 2017; Byun et al., 2018; Cui et al., 2020), PI3K inhibitor resistance (Liu et al., 2013; Jia et al., 2014; Clement et al., 2018; Tian et al., 2018; Wang et al., 2018), and vemurafenib resistance (Feng et al., 2020), et al.

Interestingly, we also observed the overexpressed S-phase kinase-associated protein 2 (Skp2) in VR1 cells when treated with different concentrations of Vem compared with parental A375 cells (Figure 1B). However, the mRNA level of Skp2 did not increase in VR1 cells (Data not shown). Additionally, in the Vem-resistant cells, the expression of Skp2 dramatically reduced to 67 and 47% after the combination treatment compared with vem treatment (lane density normalized with GAPDH, 0.8/1.2 × 100%) and VERU-111 treatment (0.8/1.7 × 100%) (Figure 2D), indicating that Skp2 plays a role in the Vem-resistance. In our experiment, we also noticed Skp2 inhibition induced by Vem (Figures 2D,E) in parental A375 cells, which might be dependent on c-Myc transcriptional regulation (Feng et al., 2020). In malignant melanoma, Skp2 is highly expressed and correlates with tumor malignancy. It is noteworthy that Skp2 E3 ligase binds to AKT and is responsible for AKT degradation, and Skp2 is also required for AKT activation and membrane recruitment (Chan et al., 2012). Conversely, phosphorylation of Skp2 on Ser72 by AKT promotes its stabilization (Song et al., 2015). In line with these studies, a dramatic reduction of AKT levels and p-AKT expression was also seen in the combination treatment group (Figure 2D). Skp2 binds with AKT (Figure 2E), and the interaction was increased in parental A375 cells while decreased in VR1 cells after Vem treatment. Collectively, the result highlighted that Skp2 is involved in Vem-resistance, and it may contribute to the synergistic effect of Vem and VERU-111. It is worthy to note that p53 expression increased upon combination treatment, which is consistent with our recent finding that VERU-111 could inhibit tumor growth and migration in cervical cancer cells by promoting DNA damage response mediated by p53 (Kashyap et al., 2020).

### Skp2 involved in mechanisms of Vem-resistance and contributes to the effect of combination treatment

**FIGURE 3:**
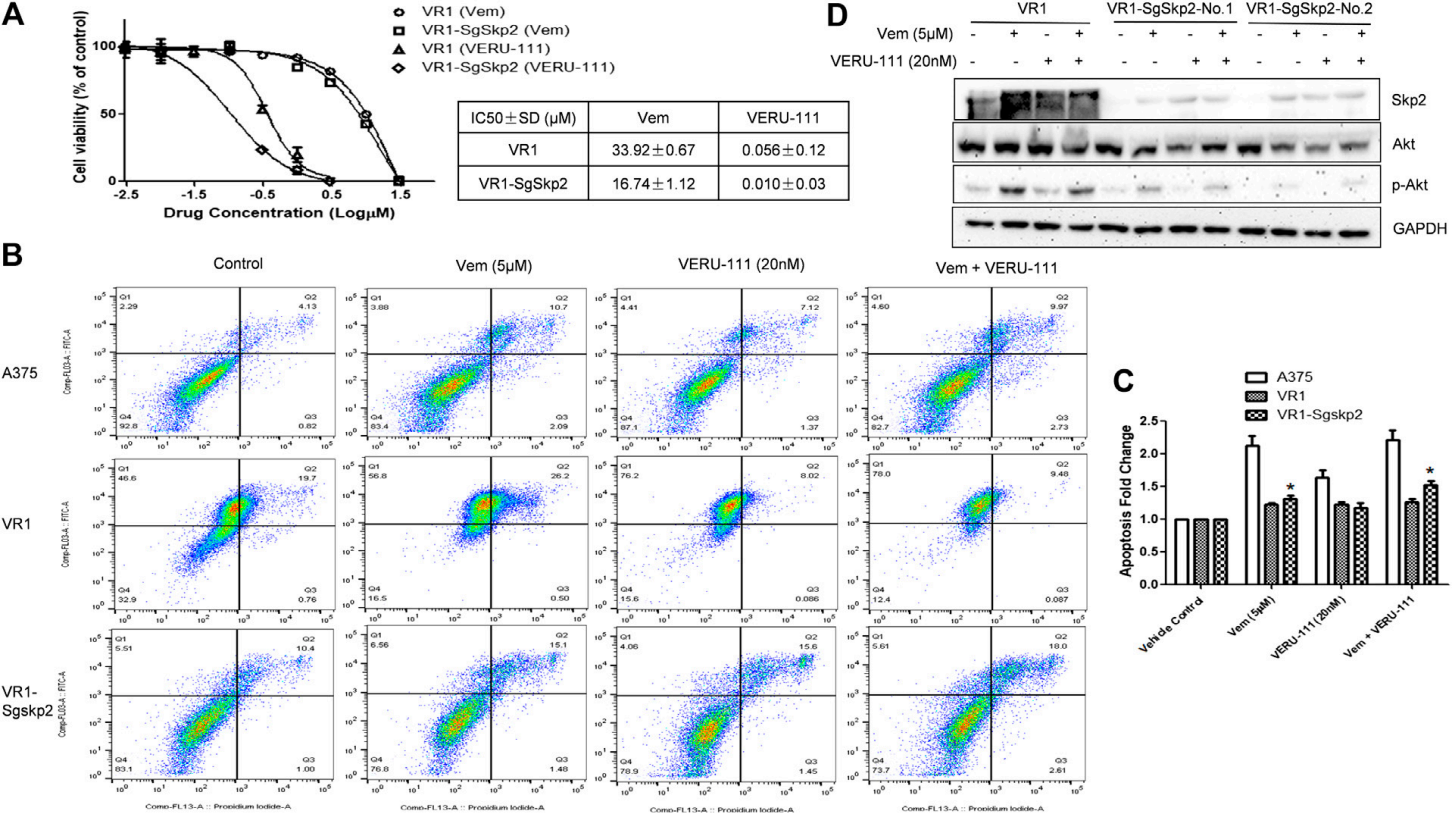
Knockout of Skp2 restore drug sensitivity of Vem-resistant cell to Vem and VERU-111 **(A)** Skp2 guide RNA (SgSkp2) was introducced to VR1 cells to make stable Skp2 knockout clones (VR1-SgSkp2). IC_50_ was measured upon indicated treatment using MTS assay **(B)** Apoptosis of VR1 and Skp2 knockout VR1 cells upon treatment. Data was analyzed by Flowjo 10.4 software. In order to compare apoptosis, we fixed gate in three cell lines, as quantified in **(C)** *, *p* < 0.05, compared with VR1 cells **(D)** Deficient of Skp2 eliminating AKT expression, which paralled with decreased p-AKT.

To further clarify the role of Skp2 in the indicated treatment, we knocked out Skp2 in VR1 cells using CRISPR-Cas9 technique. Expectedly, IC_50_ of Vem and VERU-111 improved approximately 2- and 5-fold (Vem from 33.92 to 16.74 μm and VERU-111 from 0.056 to 0.01 μm) respectively, which indicated that knockout of Skp2 not only restored compound sensitivity of VR1 cells to Vem, but also increased drug sensitivity to VERU-111 (Figure 3A). Interestingly, increased apoptosis was observed in VR1-SgSkp2 (Figure 3B). Indeed, Skp2 may inhibit apoptosis and contribute to drug resistance (Schüler et al., 2011; Wang et al., 2011). In line with these observations, we also found highly expressed Skp2 in Vem-resistant melanoma cells (Figures 1B, 2D), and decreased AKT expression and AKT phosphorylation in two single clones of VR1-SgSkp2 cells (VR1-SgSkp2-No.1 and No.2), which might be the reason to increased cell apoptosis and cell growth arrest caused by the combination regimen (Figures 2, 3C). Of note, in order to keep the resistant feature, VR1-SgSkp2 cells were still cultured in the medium with Vem, which explains minor alteration about of IC_50_ of Vem. Further analysis demonstrated that knockout of Skp2 compromised AKT activation, as indicated by decreased phosphorylation of AKT (Figure 3C).

### Combination of VEM and VERU-111 Synergistically Suppress Vemurafenib-resistant Tumor Growth *in vivo*

**FIGURE 4:**
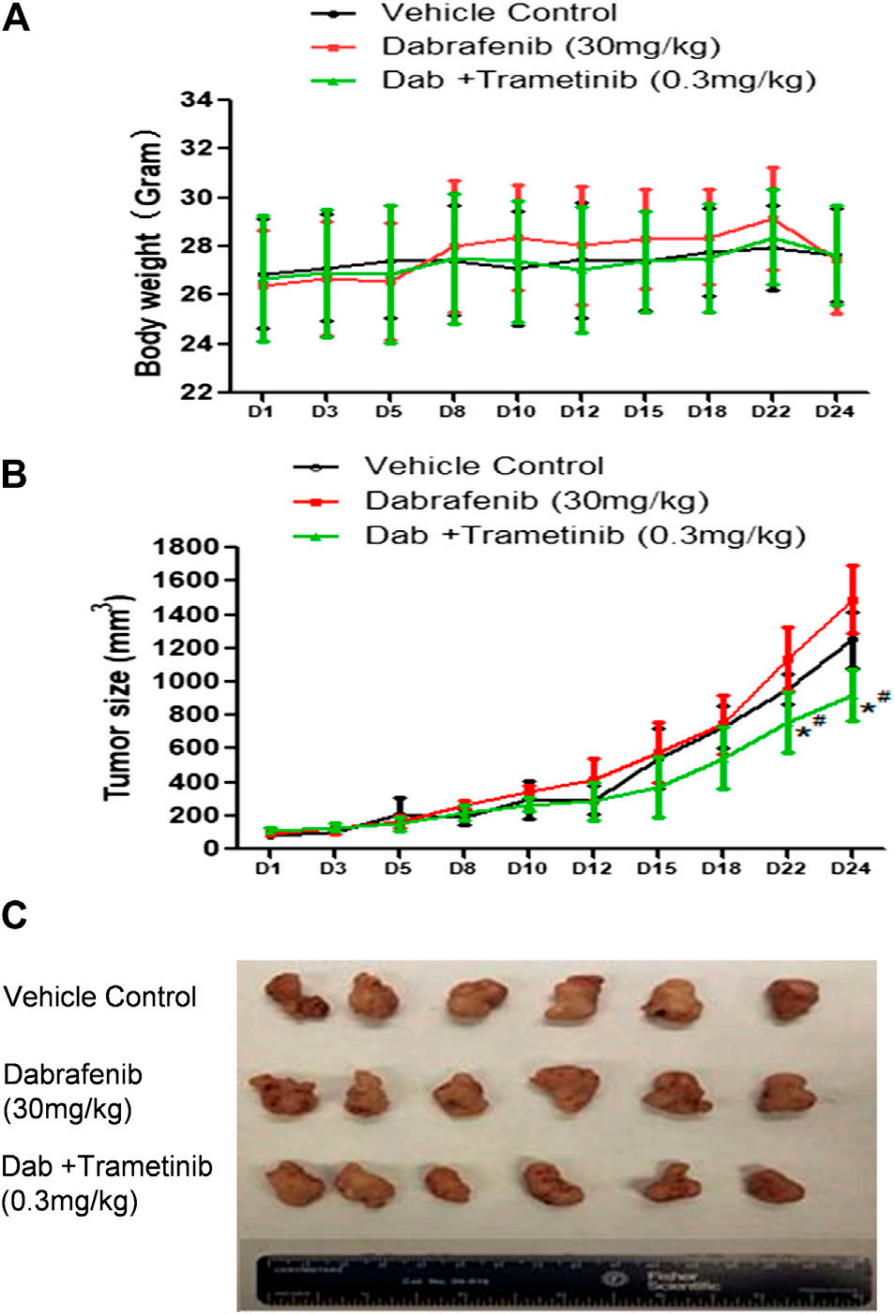
Synergistic effect of BRAF inhibitor dabrafenib and MEK inhibitor trametinib **(A)** Mice body weight curves during the administration time period **(B)** Tumor growth curve. *, *p* < 0.05, compared with Vehicle treatment groups. #, *p* < 0.05, compared with Dab treatment group. **(C)** Pictures of isolated tumor tissue.

The combination of dabrafenib (BRAF inhibitor) and trametinib (MEK inhibitor) is approved to treat Braf V600E mutant melanoma patients (Robert et al., 2015; Long et al., 2017; Hauschild et al., 2018). To evaluate our *in vivo* xenograft mouse model, we compared the inhibitory effect of dabrafebnib and its combination with trametinib. Based on previous research, the doses of 30 mg/kg dabrafenib and 0.3 mg/kg trametinib were selected (Kawaguchi et al., 2017; Yanagihara et al., 2018). Figure 4A showed no significant toxic effect in all three groups as no much change of body weight was observed. Importantly, dabrafenib plus trametinib regimen has a stronger tumor inhibitory effect (TGI at 28.6%) with statistical significance (*p* < 0.05, compared with vehicle control and dabrafenib alone) (Figures 4B,C and Table 1), demonstating the efficacy of our *in vivo* animal model.

**TABLE 1:** TGI comparison for combination of Braf inhibitor Dabrafenib and MEK inhibitor trametinib in Vem-resistant VR1 xenograft model.

Treatment group	TGI (100%)
Vehicle control	—
Dab (30 mg/kg)	121.3 ± 18.1
Dab + trametinib (0.3 mg/kg)	28.6 ± 21.2^*^

*, p < 0.05, Compared with either Dab treatment or Vehicle treatment group.

**FIGURE 5:**
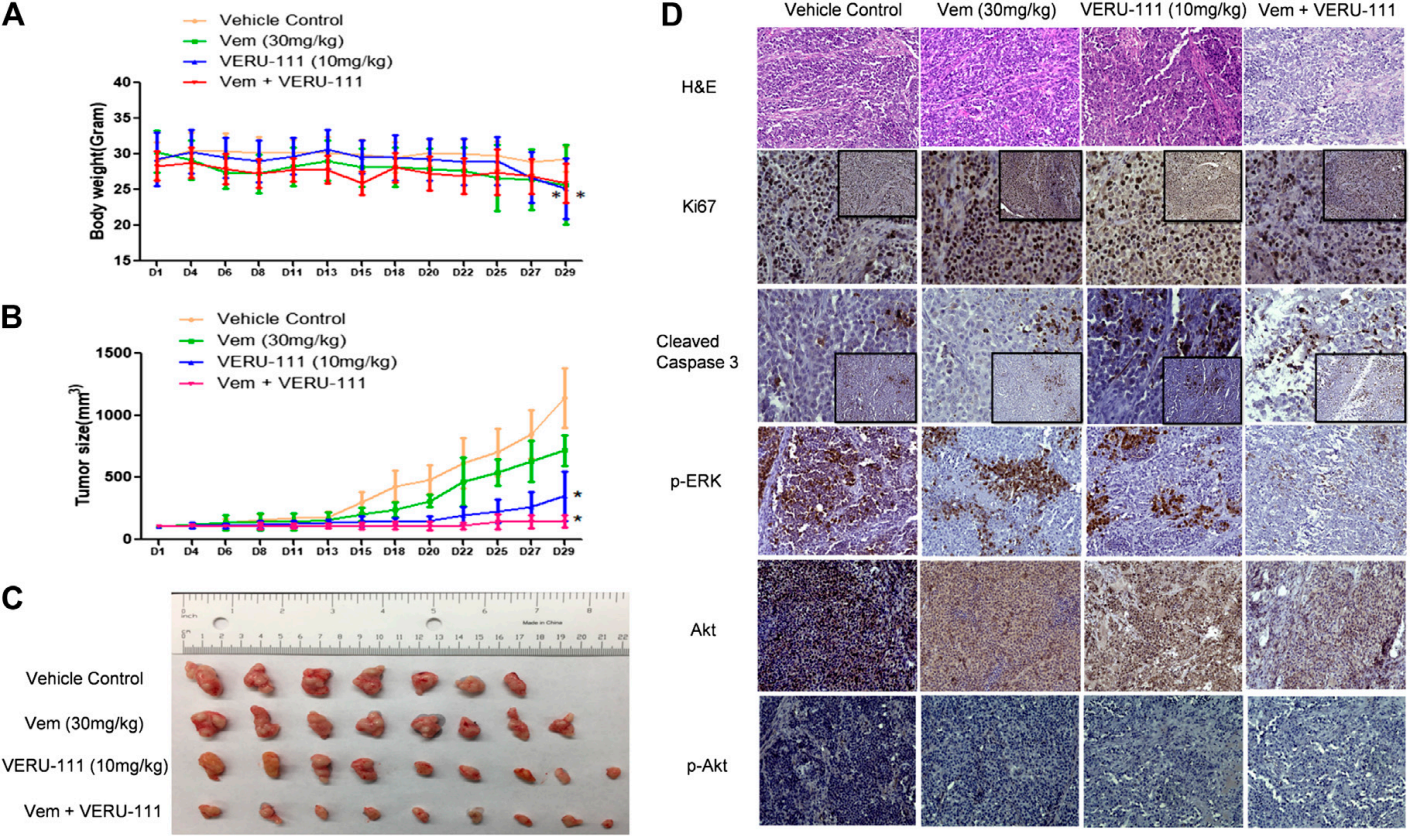
VERU-111 sensitizes VR1 tumor growth *in vivo* **(A)** Mice body weight curve with time lapse **(B)** Tumor growth curve. *, *p* < 0.05, compared with Vehicle treatment group. **(C)** Pictures of isolated tumor tissue **(D)** Representative IHC images for H&E (10×), Ki67 (10× and 40×), cleaved-caspase 3 (10× and 40×), AKT (10×), p-AKT (10×), p-ERK (10×) staining of tumor tissue sections after 4 weeks of single-agent or combination treatment.

Next, we evaluated whether there was a strong synergistic interaction between Vem and VERU-111 to counteract Vem-resistance *in vivo.* We inoculated VR1 cells in the right flank of NSG mice and treated them either with a single compound or the combination treatment strategy to assess the inhibitory effect on tumors. Based on our previous research on ABI-274, the dose of 10 mg/kg VERU-111 was selected in the current (Wang et al., 2014). As depicted in Figure 5A, no significant change was noted in body weight in all the groups. At the end of the experiment, we euthanized all the mice and examined their major organs, and no injure was found. This indicated that no general toxicity was induced by VERU-111 *in vivo*. Notably, the combination treatment strategy dramatically inhibited tumor growth compared with a single treatment or control group (Figures 5B,C), in which the tumor size in the combination group was within 100 mm^3^, while it reached 1,000 mm^3^ in vehicle group after 4 weeks of treatment. As shown in Figures 5B,C and Table 2, Vem (30 mg/kg) single treatment achieved minimal (40.6%) TGI and VERU-111 (10 mg/kg) resulted in slightly better TGI at 76.6%, whereas combination treatment significantly enhanced tumor inhibition to 96.1% after 4 weeks treatment to Vem-resistant xenograft model. Hematoxylin and eosin (H&E) staining of the tumor tissue showed that the tumor cell lost intact shape, nuclei shrank, and even some cells lost membranes, highlighting the antitumor effect of tubulin inhibitor (Figure 5D). Immunohistochemistry (IHC) staining revealed that decreased proliferation (Ki67 staining), increased cell apoptosis (cleaved-caspase three staining), and remarkably reduced expressions of *p*-ERK, total AKT and *p*-AKT (Figure 5D). Overall, the above-mentioned findings demonstrated that the tubulin inhibitor had a strong inhibitory effect on Vem-resistant tumor growth either as a single candidate or combined regimen with Vem. Additionally, VERU-111 showed a giant potential to overcome Vem-resistance in melanoma cancer cells (Figure 5D), which may be advantageous for melanoma patients harboring BRAF(V600E) mutation.

**TABLE 2:** TGI comparison for *in vivo* combination of vemurafenib (30 mg/kg) and VERU-111 (10 mg/kg) in the Vem-resistant VR1 xenograft model.

Treatment group	TGI (100%)
Vehicle control	—
Vem (30 mg/kg)	40.6 ± 11.6
VERU-111 (10 mg/kg)	76.6 ± 18.4
Vem + VERU-111	96.1 ± 4.8^*^

*, p < 0.05, compared with single-agent treatment groups.

## Discussion

Recently, the combination of BRAF inhibitor dabrafenib with MEK inhibitor trametinib was approved by FDA to treat patients harboring BRAF (V600E) mutation in NSCLC (non-small cell lung cancer) or melanoma. Although this regimen has exhibited great success in clinical therapy, patients may eventually acquire resistance after a couple of months (Robert et al., 2015; Long et al., 2017). We have developed a series of tubulin inhibitors that bind to the colchicine site in tubulin and have shown their anti-tumor effect and potential in overcoming Vem-resistance, paclitaxel-resistance in nude mice xenograft model (Lu et al., 2014; Wang et al., 2018; Wang et al., 2019; Chen et al., 2020). VERU-111 (ABI-231) is an orally available tubulin inhibitor that disrupts tubulin polymerization, promotes microtubule fragmentation, inhibits cancer cell migration, and is currently in phase 1b/2 clinical trials for men with metastatic castration and androgen-blocking agent resistant prostate cancer (ClinicalTrials.gov Identifier: NCT03752099). Tubulin inhibitor is less prone to develop resistance, therefore bearing potential to cure cancer and to sensitize drug-resistance cancer patience (Wang et al., 2014; Guan et al., 2017; Arnst et al., 2018; Deng et al., 2020; Kashyap et al., 2020; Mahmud et al., 2020).

In this study, we investigated whether the orally derivative of ABI-274, VERU-111, has synergistic effect with Vem. VERU-111 has been tested in many cancer cell lines and its IC_50_ is 5.6 nM in M14 cell, 7.2 nM in WM164 melanoma cell (Wang et al., 2019), 8.2 nM in MDA-MB-231 breast cancer cell (Chen et al., 2020), 55.6 nM in NSCLC A549 cell and 102.9 nM in A549-Paclitaxel resistant cells (Mahmud et al., 2020). In agreement with outcomes of previous research, it was confirmed that by synergisticly arresting cancer cells at G0-G1 and G2-M phases, the combined treatment regimen of Vem and VERU-111 could overcome the Vem-resistance through enhanced apoptosis and compromised Skp2-AKT signaling pathway. In a tumor xenograft model, the combined regimen displays a better inhibitory efficiency against tumor progression than either single treatment. Further IHC analysis of tissue sections confirmed decreased tumor proliferation and the diminished expression of AKT and *p*-AKT. Several studies reported an association between inhibition of AKT and tubulin polymerization (Zhang et al., 2009; Krishnegowda et al., 2011; Viola et al., 2012). Inhibition of AKT-mediated survival signaling pathway has been shown to increase sensitivity to microtubule-targeted tubulin-polymerizing agents (MTPAs)-induced apoptosis in cancer cells (Bhalla 2003). The results of the present research are consistent with findings of these studies, highlighting a close interaction between tubulin polymerization inhibitors and downregulation of AKT in melanoma.

Remarkably, Skp2 E3 ligase was also involved in the mechanisms of Vem-resistance and synergistic effect of combination regimen. Recent studies reported that overexpressed Skp2 was found in paclitaxel-resistant prostate cancer cells (Yang et al., 2016; Byun et al., 2018; Gong et al., 2018), and knockdown of Skp2 restored the sensitivity of paclitaxel in prostate cancer cells (Byun et al., 2018). Skp2 also plays a pivotal role in mitosis and spindle checkpoint by triggering ubiquitination and activation of Aurora-B (Nakayama et al., 2004; Sugihara et al., 2006; Hu and Aplin, 2008; Wu et al., 2015). Skp2 depletion in melanoma cells resulted in a G2-M phase arrest (Hu and Aplin 2008), and suppression of both BRAF (V600E) and Skp2 inhibited cell growth and invasion in melanoma cell lines(Sumimoto et al., 2006). Since Skp2 was reported to interact with AKT (Chan et al., 2012), we also tested the interaction and found decreased AKT expression and AKT phosphorylation in VR1-SgSkp2 cells (Figures 2E, 3D), thereby leading to cell apoptosis and cell growth arrest caused by the combination treatment (Figure 3). Meanwhile, BRAF inhibitor dabrafenib combination with MEK inhibitor trametinib present a mild synergistic effect in inhibition of tumor growth, as shown in Figure 4 and Table 1. By contrast, our *in vivo* xenograft tumor model demonstrated that combination regimen of Vem and VERU-111 has more potent tumor inhibitory effect than single administration (Figure 5 and Table 2). When aministrtaed in combination with Vem, VERU-111 has a tumor growth inhibitory rate (TGI) of 96.1%, which was better than ABI-274 (TGI 88.6%) [Table 2 and reference (Wang et al., 2014)].

Collectively, based on our study, VERU-111 overcome Vem-resistance through the following mechanisms: 1) As a tubulin destabilizing agent, disrupt tubulin polymerization, promote microtubule fragmentation, inhibit cancer cell migration; 2) Combined with Vem, arresting cell both in G0-G1 and G2-M phase; 3) Compromised Skp2-AKT signaling pathway. Our study showed that VERU-111bears inspiring potential in synergistically combination with BRAF inhibitor Vem to overcome drug resistance in melanoma. Furthermore, this synergistic effect might through regulating Skp2-AKT, as evidenced by increased apoptosis and drug sensitization when skp2 was knocked out, which suggested that silencing skp2 might be an effective way to overcome Vem-resistance.

## Conclusion

In conclusion, our findings provide direct evidence and a reasonable explanation for giving a combination of a tubulin inhibitor VERU-111 with a BRAF inhibitor to overcome Vem-resistance in melanoma pateints.

## Data Availability

The original contributions presented in the study are included in the article/Supplementary Material, further inquiries can be directed to the corresponding author.
